# On the microstructurally driven heterogeneous response of brain white matter to drug infusion pressure

**DOI:** 10.1007/s10237-022-01592-3

**Published:** 2022-06-18

**Authors:** Tian Yuan, Wenbo Zhan, Asad Jamal, Daniele Dini

**Affiliations:** 1 Department of Mechanical Engineering, Imperial College London, London, SW7 2AZ UK; 2 School of Engineering, King’s College, University of Aberdeen, Aberdeen, AB24 3UE UK

**Keywords:** Permeability, Porosity, Heterogeneous response, Brain tissue, Convection-enhanced delivery, Multiscale modelling

## Abstract

Delivering therapeutic agents into the brain via convection-enhanced delivery (CED), a mechanically controlled infusion method, provides an efficient approach to bypass the blood–brain barrier and deliver drugs directly to the targeted focus in the brain. Mathematical methods based on Darcy’s law have been widely adopted to predict drug distribution in the brain to improve the accuracy and reduce the side effects of this technique. However, most of the current studies assume that the hydraulic permeability and porosity of brain tissue are homogeneous and constant during the infusion process, which is less accurate due to the deformability of the axonal structures and the extracellular matrix in brain white matter. To solve this problem, a multiscale model was established in this study, which takes into account the pressure-driven deformation of brain microstructure to quantify the change of local permeability and porosity. The simulation results were corroborated using experiments measuring hydraulic permeability in ovine brain samples. Results show that both hydraulic pressure and drug concentration in the brain would be significantly underestimated by classical Darcy’s law, thus highlighting the great importance of the present multiscale model in providing a better understanding of how drugs transport inside the brain and how brain tissue responds to the infusion pressure. This new method can assist the development of both new drugs for brain diseases and preoperative evaluation techniques for CED surgery, thus helping to improve the efficiency and precision of treatments for brain diseases.

## Introduction

Brain diseases, such as Parkinson’s disease, Alzheimer’s disease, and malignant brain tumour, have been laying considerable burden on global health and economics in the recent decades (Feigin et al. 2020). It is estimated by WHO that the death due to brain diseases will reach 12.22% of total death in 2030 worldwide (Feigin et al. 2019). Although some effective drugs for brain diseases have been clinically approved (Group 2004; Pinheiro and Faustino 2019), it is still challenging to deliver them into the brain properly. The main reason is that the blood–brain barrier (BBB), a highly selective border surrounding most of the blood vessels of the brain, blocks 100% of the macromolecular and over 98% of the small-molecular drugs from entering into the brain parenchyma (Bors and Erdö 2019).

**Fig. 1:**
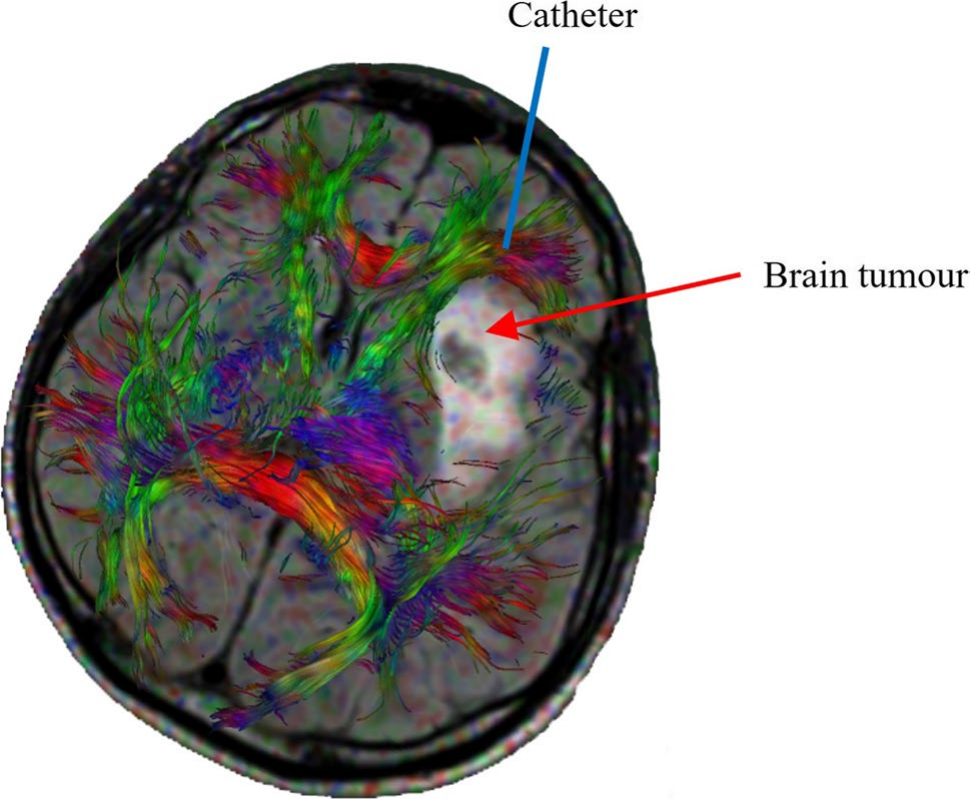
Schematic of convection-enhanced delivery and diffusion tensor image of a brain with a tumour. The colourful bundles are nerve fibres. This figure is adapted from Ref. (Zelenak et al. 2013) with open access under the terms of the Creative Commons Attribution 3.0 License

Convection-enhanced delivery (CED) (Hunt Bobo et al. 1994), a mechanically controlled infusion method that delivers therapeutic agents directly to the target regions through one or more implanted catheters, therefore, is regarded as a promising technique for brain diseases treatments (Mehta et al. 2017). The schematic of CED is shown in Fig. 1. However, making the appropriate preoperative planning, such as the choice of injection location, infusion pressure, and the size of catheter is very difficult, because it is technically challenging to make precise prediction of drug transport in brain tissue (Raghavan et al. 2006). As a result, few desired results have been achieved in clinical trials of CED (Lang et al. 2006; Salvatore et al. 2006; Sampson et al. 2010). The fundamental reason can be, in fact, attributed to the insufficient understanding of (i) brain tissue’s transport properties and (ii) brain tissue’s mechanical response to the infusion pressure, which further alters the intrinsic transport properties of brain tissue.

Transport properties and mechanical responses to infusion pressure of brain tissues are fundamentally determined by the tissue’s microstructure (Chen and Sarntinoranont 2007). Brain is mainly composed of neuronal cells, with the cell bodies forming grey matter and the nervous fibres (axons) constituting WM. WM can be regarded as a bridge between different brain regions because axons play the role of transmitting information among different functional cells, such as neurons, muscle, and gland cells (Fields 2008). To this end, axons in WM are distributed as bundles, which have specific orientations (Sperber 2006). Figure 1 displays an output of diffusion tensor imaging (DTI) showing a brain with a tumour, where the distribution and orientations of the axons (the colourful fibres) in WM are clearly visible. Given the presence of these fibre bundles, WM is normally described as an anisotropic porous medium, the transport properties of which are characterized by porosity and hydraulic permeability (Linninger et al. 2008). Drug transport in WM, therefore, can be modelled as porous flow and predicted mathematically.

In order to consolidate the fundamental theories and practical procedures of CED, many mathematical simulations have been done to understand the effects of individual factors. For example, methods based on Darcy’s law (Brady et al. 2011; Stine and Munson 2019) and computational fluid dynamics (Arifin et al. 2009; Vidotto et al. 2019) were developed to investigate the effects of the geometry of the catheter (Brady et al. 2018), the anisotropy of the brain tissue (Zhan et al. 2019), and the properties of the agents (Zhan and Wang 2018) on the drug delivery efficiency in brain. In most of these simulations, brain microstructure was assumed to be rigid, thus the porosity and hydraulic permeability were uniform, homogeneous, and independent to the infusion pressure.

If drug transport is driven by diffusion, these assumptions are indeed valid in some situations. However, in CED, the increased local hydraulic pressure would necessarily deform the local microstructure because the WM tissue components are extremely soft in nature (Bernal et al. 2007). In fact, even without considering CED, it has been reported that intracranial pressure has significant individual differences and varies with time and age. While the normal average intracranial pressure is 933.3–1999.8 Pa in adults in a horizontal position and 252.7 Pa in a vertical position, it has a fluctuation of 558.6 Pa for different people, a decrement of 91.8 Pa per decade (Pedersen et al. 2018). These background pressure variations may also be significant enough to change the microstructure of brain tissue. Therefore, the assumptions of homogeneous and pressure-independent porosity and permeability would inevitably introduce errors to the prediction of drug transport in brain tissue, especially when the infusion pressure is high in CED (normally ~ 4000 Pa (Hunt Bobo et al. 1994)). A good example is provided by our recent experimental work, which investigated the effect of axonal bundles orientation on the transport properties of WM through controlled infusion in brain tissue, where a nonlinear relationship between infusion pressure and hydraulic permeability of the brain tissue was found (Jamal et al. 2021). Therefore, these two parameters should be modelled in a location-dependent and pressure-dependent manner within the tissue domain due to the non-uniformly distributed hydraulic pressure.

Indeed, there are models being developed to consider linear and nonlinear changes of permeability due to deformations, for example the biphasic or poroelastic models for brain tissues (Chen and Sarntinoranont 2007; Smith and García 2009; García and Smith 2009), cartilage (Mattei et al. 2014; Behrou et al. 2018), and other soft tissues (Wu and Herzog 2002). The fundamental formulations of linear and nonlinear poroelastic theories and their links have been overviewed and thoroughly discussed in Macminn et al. (2016). In fact, permeability is not only pressure dependent, but it also varies spatially and temporally because of the local variation of applied pressure gradients. To describe this type of porous-media behaviour, Barrera’s group introduced Caputo fractional derivatives into Darcy’s law, and implemented this fractional pore pressure diffusion equation in ABAQUS (Barrera 2021). This modelling scheme was then adopted to successfully predict the human meniscus behaviour under confined compression conditions against experiments (Bulle et al. 2021). However, these methodologies most often adopted poroelastic theory, and thus integrating the solid and fluid phases in a continuum model (Jamal et al. 2022b). Although the solid deformation and fluid flow inside the continuum media can be explicitly solved, obtaining the deformation-driven permeability relies on some empirical or semi-empirical formulas that link permeability to porosity. The most adopted formula is Kozeny–Carman (KC) equation, but the KC constant is an empirical parameter and highly depends on the microstructure of the porous media (Xu and Yu 2008). Therefore, an explicit physics-based connection between the microscopic tissue deformation and the macroscopic transport property change of the tissue is very important to explore but has escaped researchers so far.

To fill this gap, a multiscale model based on Darcy’s law and fluid–solid interaction (FSI) was established in this paper. While Darcy’s law is adopted in the macroscopic model to evaluate the global pressure distribution, FSI is used to model the interactions between axons and interstitial fluid (IF) at the microscopic level to characterize the local permeability and porosity of the tissue domain and their variation as a function of the local hydraulic pressure. By exchanging the information between the micro- and macroscopic models, the multiscale framework was formed. It was then tested against the results obtained through infusion experiments in ovine brain (Jamal et al. 2021). The newly established multiscale model not only successfully explained the mechanism behind the nonlinear response of brain WM to infusion pressure observed in the experiments, but it constitutes a framework that can be enriched by adding more complex descriptions of the nonlinear behaviour or the tissue components at microstructural level and can also be used to provide more accurate predictions and enhance strategies for drug delivery in the brain.

## Methodology

### Governing equations: macroscopic model

Darcy’s law, as shown in Eq. (1), is a simplified and homogenized form of Navier–Stokes equation with the neglection of gravitational forces (Whitaker 1986); it is valid only for small Reynolds numbers when inertial forces are small compared to viscous forces. Equation (2) is the continuity equation of porous media, where $\phi$ denotes the porosity, as expressed in Eq. (3). By solving Eqs. (1) to (3), the fluid filed in a given porous medium could be obtained by measuring the fluid discharge rate once the properties of the fluid and the porous media are known.

1 $${v}_{D}=\frac{Q}{A}=\frac{K}{\mu }\frac{\Delta p}{L}$$

2 $$\begin{array}{c}\frac{\partial}{\partial t}\left({\rho}_{f}\phi \right)+\nabla \cdot \left({\rho }_{f}{\mathbf{v}}_{D}\right)=0\end{array}$$

3 $$\begin{array}{c}\phi =1-\frac{{V}_{\rm axons}}{{V}_{\rm sample}}\end{array}$$

where $Q \left(\mathrm{SI unit}:{\rm m}^{3}/{\rm s}\right)$ is the fluid discharge rate; $K \left({\rm m}^{2}\right)$ is the apparent hydraulic permeability of the whole sample; $A$ is the cross-sectional area of the sample; $L$ is the length of the sample; $\Delta p$ is the pressure drop along the sample; $\mu$ and ${\rho }_{f}$ are the dynamic viscosity, density and velocity vector of the IF, respectively; $\phi$ is the apparent porosity of the domain; ${V}_{\rm sample}$ is the total volume of the sample while ${V}_{\rm axons}$ is the volume of the axons inside the given sample. ${\mathbf{v}}_{D}$ is the superficial or Darcy velocity, which is an equivalent flow velocity (i.e. velocity of fluid passing through per unit cross-sectional area of the domain). If the local velocity inside the pores is denoted as ${\mathbf{v}}_{f}$, then ${\mathbf{v}}_{D}=\phi {\mathbf{v}}_{f}$. It is worth mentioning that all the variables in Eqs. (1–3) are equivalent variables for the whole sample.

### Governing equations: microscopic model

In reality, interactions between fluid flow and soft tissues/membranes are very common inside human body and have been widely studied by FSI simulations, such as FSI between blood flow and blood vessel (Keramati et al. 2020), bone marrow and trabecular bone (Rabiatul et al. 2021). Therefore, a microscopic model based on FSI was developed here to quantify how much the pressure-driven deformation of the axons affects the local permeability and porosity of the brain tissue, to be provided as input to improve the otherwise inaccurate macroscopic description of fluid flow in the tissue.

In the microstructure, the drug solution is modelled as isothermal Newtonian flow that is governed by Navier–Stokes equations, as shown in Eqs. (4) and (5).

4 $$\begin{array}{c}\frac{D\rho }{\rm Dt}+\nabla \cdot {\mathbf{v}}_{f}=0\end{array}$$

5 $$\begin{array}{c}{\rho }_{f}\frac{\partial {\mathbf{v}}_{f}}{\partial t}+{\rho }_{f}\left({\mathbf{v}}_{f}\cdot \nabla \right){\mathbf{v}}_{f}=\nabla \cdot {{\varvec{\upsigma}}}_{f}+{\rho }_{f}{\mathbf{F}}_{f}\end{array}$$

where ${p}_{f}$ is pressure, ${{\varvec{\upsigma}}}_{f}$ is the stress tensor of the IF, as given in Eq. (6), with $\mathbf{I}$ being the identity matrix. ${\mathbf{F}}_{f}$ is the volume force vector of unit mass, which we did not consider here.

6 $$\begin{array}{c}{{\varvec{\upsigma}}}_{f}=-{p}_{f}\bf{I}+\mu \left[\nabla {\mathbf{v}}_{f}+{\left(\nabla {\mathbf{v}}_{f}\right)}^{T}\right]-\frac{2}{3}\mu \left(\nabla \cdot {\mathbf{v}}_{f}\right).\end{array}$$

It is worth mentioning that the Reynolds number of the infusion flow is usually so low, at the level of 1E-5 (Hunt Bobo et al. 1994), that the flow can be regarded as Stokes flow. In addition, it was assumed that the IF is incompressible. Therefore, the first term in Eq. (4) and the inertial term of Eq. (5) can be neglected, so Eqs. (4)–(6) are simplified to be:

7 $$\begin{array}{c}\nabla \cdot {\mathbf{v}}_{f}=0\end{array}$$

8 $$\begin{array}{c}{\rho }_{f}\frac{\partial {\bf{v}}_{f}}{\partial t}=-\nabla {p}_{f}\bf{I}+\mu {\nabla }^{2}{{\bf{v}}}_{f}+{\rho }_{f}{\bf{F}}_{f}\end{array}$$

The structural deformation is driven by the fluid pressure and is governed by Eq. (9).

9 $$\begin{array}{c}{\rho }_{s}\frac{{\partial }^{2}{\mathbf{u}}_{s}}{\partial {t}^{2}}=\nabla \cdot {{\varvec{\upsigma}}}_{s}+{\rho }_{s}{\mathbf{F}}_{s}\end{array}$$

where ${\rho }_{s}$ is the density of the axon, ${\mathbf{u}}_{s}$ is the displacement vector of the axon, ${{\varvec{\sigma}}}_{s}$ is the stress tensor of the solid, and ${\mathbf{F}}_{s}$ is the volume force vector of unit mass, such as gravity. Here, we did not consider volume force, so ${\mathbf{F}}_{s}=0$.

Considering the potentially large deformation of the axons, the Arbitrary Lagrange–Eulerian (ALE) framework together with moving mesh method were used to capture the two-way interaction between the fluid phase and solid phase. The first step is to solve the fluid flow (Eqs. 7 and 8) and calculate the reaction force on the IF. Then, the force is applied on the axon wall on the basis that the components of the reaction force on IF and the load on axons in the direction of outward normal to the solid boundary are equal, as shown in Eq. (10). Next, the deformation of the axons can be calculated by solving Eq. (9), and the solid displacement is used to update the geometry and mesh of fluid domain while the velocity of the solid boundary is applied to the fluid, as governed by Eqs. (11) and (12). Note the assumptions here are no slip and no gap between the solid phase and fluid phase. This is the one loop of the two-way FSI. More loops will be carried out until the converged solution be achieved.

10 $$\begin{array}{c}{{\varvec{\upsigma}}}_{s}\cdot \bf{n}={{\varvec{\upsigma}}}_{f}\cdot \bf{n} \end{array}$$

11 $$\begin{array}{c}{\mathbf{u}}_{s}={\mathbf{u}}_{\rm mesh}\end{array}$$

12 $$\begin{array}{c}\frac{\partial {\mathbf{u}}_{s}}{\partial t}={\mathbf{v}}_{\rm wall}={\mathbf{v}}_{f}\end{array}$$

where $\mathbf{n}$ denotes the normal direction; ${\mathbf{u}}_{\mathrm{mesh}}$ and ${\mathbf{u}}_{s}$ are the displacement of the mesh and the axon’s wall, respectively; ${\mathbf{v}}_{\rm wall}$ is the velocity of the axon’s wall.

### Geometries: macroscopic model

**Fig. 2:**
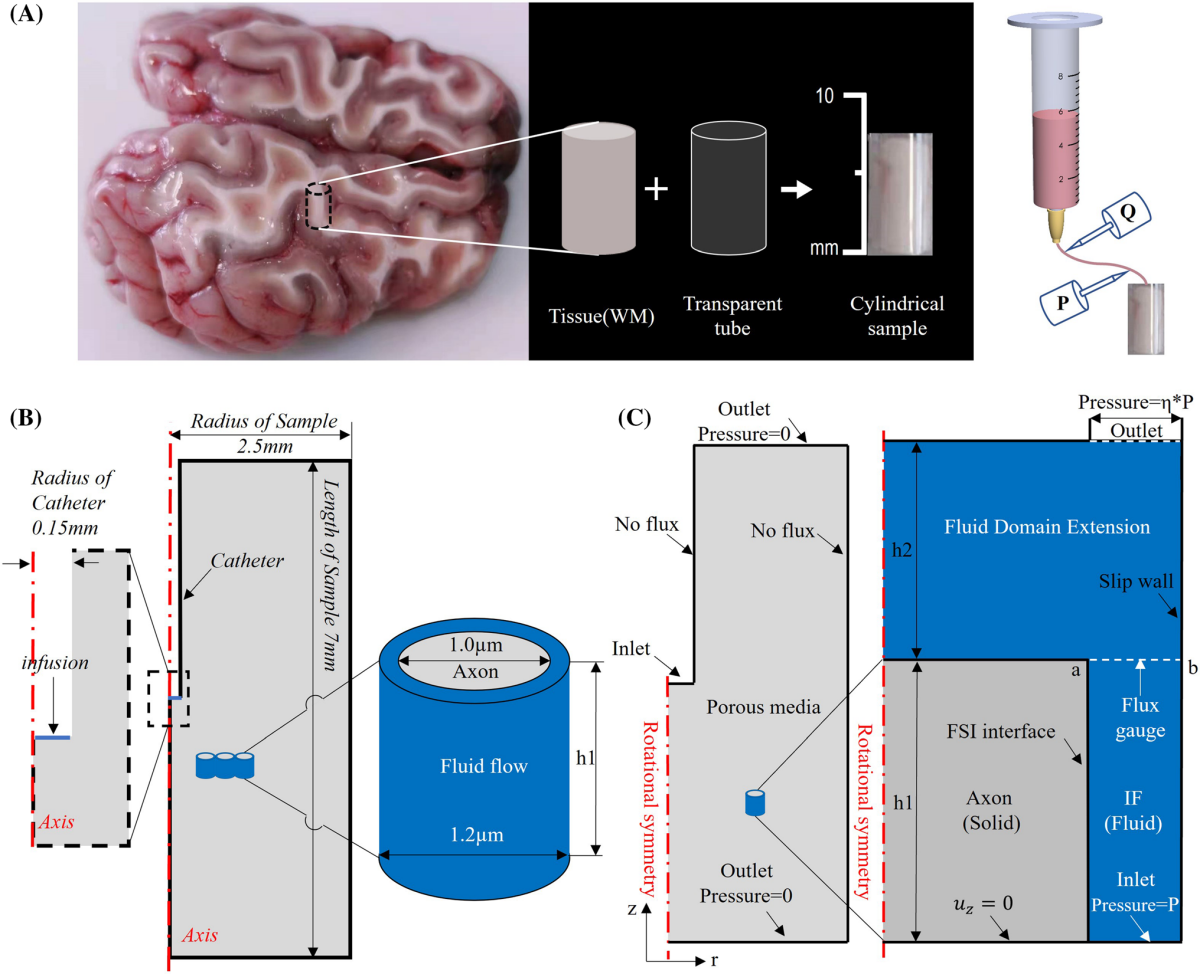
**a** Schematic of sampling and experimental methods. **b** Schematic of the multiscale model and the dimensions. Middle: Section view of the macroscopic model (cylinder). Left: Enlarged view of drug injection point. Right: Microscopic model. **c** Boundary conditions for Left: Macroscopic model. Right: microscopic model.  ${u}_{z}$=0 means z displacement is fixed. In both figures (**b**) and (**c**), the grey part is axon (solid) and the blue part is IF (fluid)

The macroscopic model comes from the experimental sample, which is cylindrical with a diameter of 5 mm and a length of 7 mm. A needle with an inner diameter of 0.3 mm was injected into the sample with a depth of 3.5 mm for infusion (Fig. 2). The schematic of sampling and experimental methods is shown in Fig. 2a.

**Fig. 3:**
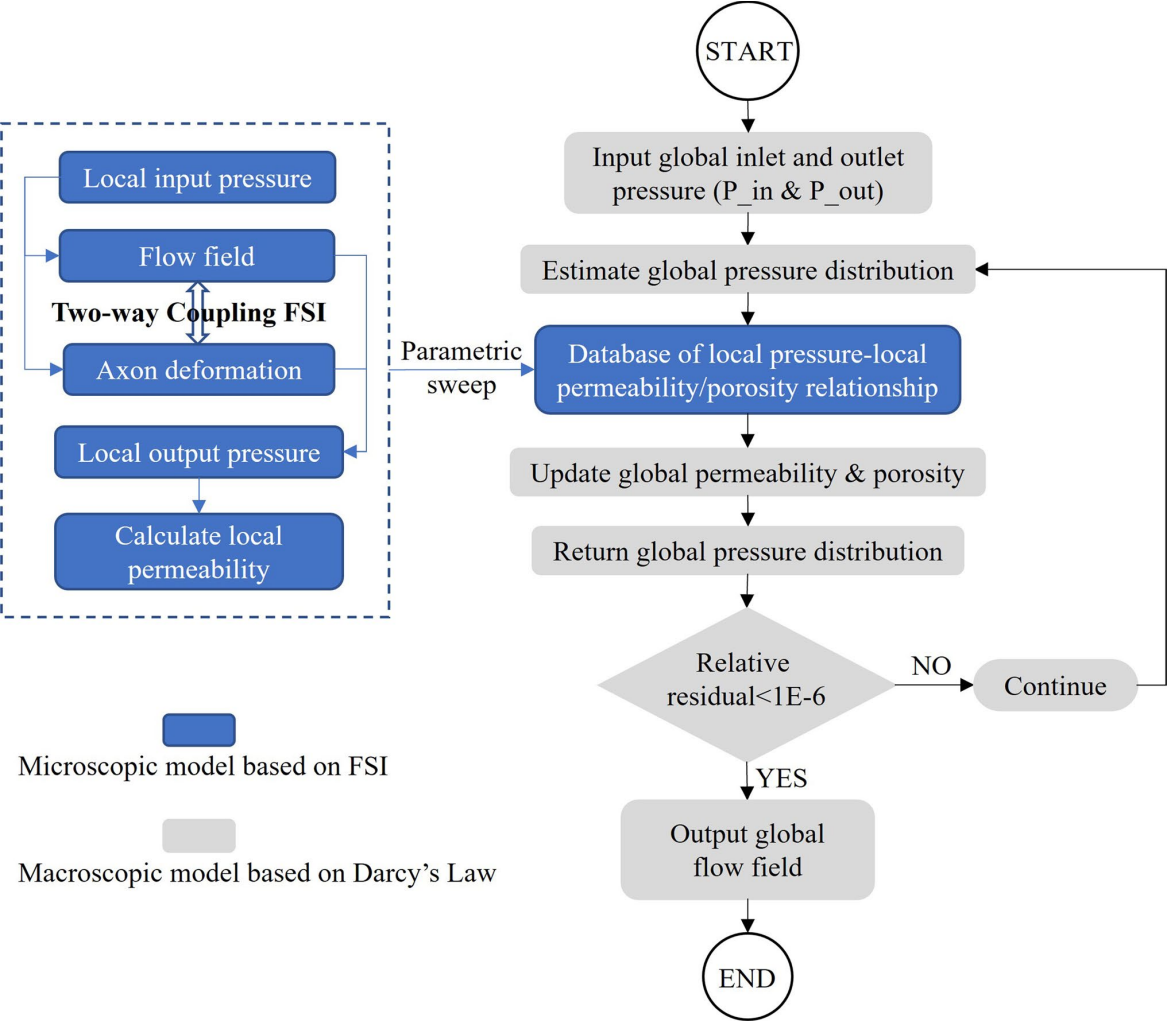
Flow chart of the information exchange inside the multiscale model

### Geometries: microscopic model

Since the microscopic model is used to characterize the local permeability and porosity of the brain tissue, it should be able to represent the microstructure of the tissue. According to the database from the literatures, the average diameter of axons is 1 ${\upmu} \mathrm{m}$ (Liewald et al. 2014), while the porosity ($\phi$) of brain tissue does not exceed 0.3 (Vidotto et al. 2019). Therefore, in the microscopic model, the radius of the axon and the sample is set as 0.5 ${\upmu} \mathrm{m}$ and 0.6 ${\upmu} \mathrm{m}$, respectively. The choice of the representative axon diameter is discussed in "Appendix A". The effective length of the microscopic model h1 was set as 1 ${\upmu} \mathrm{m}$. However, due to the ductility of the axon, the axon will swell in the axial direction when being compressed in the radial direction. In order to free its axial deformation while preserving the quality of the mesh, in particular at the outlet, the computational domain of the fluid is elongated at the length of h2 = 1.3 ${\upmu} \mathrm{m}$ along the direction of the outflow. h1 and h2 were determined by sensitivity studies, as described in "Appendix B". In fact, this treatment also has its own limitations, as in the real tissue, each element of an axon of length h1 is in contact with another element of axon instead of a fluid domain, therefore the elongation of the axon might be overestimated in this model. The multiscale model and the computational domains are illustrated in Fig. 2b, c.

### Boundary conditions: macroscopic model

Fig. 2C shows the boundary conditions of the multiscale model. For the macroscopic model (left-hand portion), inlet and outlet boundaries were controlled by pressure. While the inlet pressure was variable to study the effect of infusion pressure, the outlet pressure was set as 0 Pa. It was assumed that there is no flow across the faces that contact with the transparent tube (see Fig. 2a), which is governed by:

13 $$\begin{array}{c}\bf{n}\cdot {\mathbf{v}}_{D}=0\end{array}$$

where **n** denotes the normal direction.

### Boundary conditions: microscopic model

For the microscopic model, there are some key features that need to be discussed as follows:

1. In order to maintain the prediction accuracy of permeability while the fluid domain is elongated, flux at the edge labelled as “ab” in the right-hand portion of Fig. 2c is monitored and used to calculate of the local permeability.
2. Aiming at obtaining the local permeability and porosity of the tissue for specific values of pressure, the pressure of the fluid domain should be uniform. However, a very small pressure gradient is needed to establish flow through the fluid domain, which was implemented by setting the outlet pressure to be *η* × inlet pressure, where *η* should be very close to 1. *η* was also determined by a sensitive study as reported in Appendix B.
3. Permeability in white matter is anisotropic in nature because of the directionality of the axons (Jamal et al. 2022a) and should therefore be described as a tensor. However, in this study our focus is to obtain a better understanding of the microstructural origin (axonal deformation) of the macroscopic response of the tissue to infusion pressure, which results in permeability changes. This is achieved by building the proposed multiscale modelling tool and testing its validity and implications of the results by exploring, for simplicity, infusion in the direction parallel to the axons. Therefore, using the full description of the permeability tensor in the macroscale model was deemed unnecessary in this work. Here, the parallel component of the permeability tensor was used in the macroscale model as representative of the behaviour of the material, which was treated as isotropic. While this is an approximation, our choice does not affect the comparison between the models used to describe the microscopic behaviour of the tissue and does not change the main findings in terms of the effect that pressure has on permeability.
4. The boundary conditions at the right edge of the fluid domain are governed by Eqs. (14) and (15), which means the fluid flow cannot penetrate the boundary (Eq. (14)) while, at the same time, there are no viscous effects on this wall and no boundary layers developed (Eq. 15). From a modelling point of view, this is to consider the fact that the outside of this microscopic model corresponds to the presence of IF instead of axons. Note that the gravity was not included.
  14 $${\mathbf{n}}\cdot {\mathbf{v}}_{f}=0$$
  15 $$\begin{array}{c}\left\{-{p}_{f}\mathbf{I}+\mu \left[\nabla {\mathbf{v}}_{f}+{\left(\nabla {\mathbf{v}}_{f}\right)}^{\rm T}\right]\right\}\cdot \bf{n}=0 \end{array}$$

### Information exchange between the models at separate scales

Figure 3 shows the flow chart that interprets the information exchange inside the multiscale model. Firstly, using the microscale model, parametric studies were conducted to establish a database where the local tissue permeability and porosity correspond to a specific value of local fluid pressure. The range of the fluid pressure was set as 0 Pa—3 kPa based on the experimental infusion scenarios considered here. The local permeability and local porosity were then calculated by Eqs. (16–17).

16 $$\large \begin{array}{c}k=\frac{{v}_{f} \left({r}_{m}^{2}-{r}_{a}^{2}\right)\mu {h}_{1}}{{r}_{m}^{2} P(1-\eta )}\end{array}$$

17 $$\large \begin{array}{c}\varphi =1-\frac{{r}_{a}^{2}}{{r}_{m}^{2}}\end{array}$$

where $k$ is the local permeability and $\varphi$ is the local porosity, which should be distinguished from the equivalent permeability ($K$) and equivalent porosity ($\phi$) used for the whole domain in Eqs. (1–3); ${{r}}_{\mathrm{a}}$ is the radius of deformed axon and ${{r}}_{\mathrm{m}}$ is the radius of the microscopic model (0.6 µm); $\mathrm{P}$ is the inlet pressure of the microscopic model.

Secondly, subject to the given boundary conditions, the macroscale model was applied to generate the initial fluid field in the entire domain. The fluid pressure at each node is then used to update the local tissue permeability and porosity, based on the database obtained from microscale model. The above steps work iteratively, and the simulation needs to run several iterations until the residual is below the given tolerance, which was set as 1E-6.

It is worth mentioning that the permeability and porosity database built by solving the microscopic model should be a function of both local pressure and pressure gradient in the real situation. However, since we only consider the parallel direction in the present study, and the axonal length (h1) is only 1 µm which makes the axon can be treated as a straight column, the radial compression dominates the axonal deformation/displacement. In addition, as the pressure gradient is very low as discussed in Sect. 2.6 (2), it has very limited effect on the axonal radial compression. Therefore, the variable of pressure gradient can be eliminated in the present function.

### Material properties

The properties of the materials in this multiscale model and their sources are shown in Table 1. It should be noted that the viscosity used here is the viscosity of IF, instead of the viscosity of drug fluid. In this paper, the constitutive behaviour of axons was described as linear elastic (Zarei et al. 2017); this is not a limiting assumption for the present work, which focuses on the establishment of a multiscale modelling framework and allows for more complex nonlinear constitutive laws to be considered to describe the mechanical response of axons under load at a later stage.

**Table 1:** Material properties

Parameters	Unit	Value	Description	Source
E	Pa	12,000	Young’s modulus of the axon	Bernal et al. (2007)
*υ*	–	0.49	Poisson’s ratio of the axon	Ouyang et al. (2013)
${\rho }_{\mathrm{s}}$	kg/m^3^	1050	Density of the axon	Chen et al. (2019)
*μ*	Pa s	3.5E-3	Dynamic viscosity of IF	Yao et al. (2013)
${\rho }_{\mathrm{f}}$	kg/m^3^	1000	Density of IF	Yao et al. (2013)
$\phi$	–	0.3	Porosity of WM	Vidotto et al. (2019)

## Numerical results and experimental validation

The mathematical models were solved in the software package COMSOL Multiphysics 5.6 (COMSOL Multiphysics® 2021). Structural elements were used in both macroscopic and microscopic models. Benefited from axial symmetry of both models, the element size could be very fine to guarantee the accuracy, so element sizes of 0.05 mm and 0.01 µm were used in the macroscopic model and the microscopic model, respectively, after mesh sensitive tests. MUMPS (MUltifrontal Massively Parallel sparse direct Solver) was adopted for both microscopic and macroscopic simulations to accelerate the computation speed.

In this section, results at different scales will be first analysed to see how microscopic FSI between axon and IF affect the macroscopic material behaviours. Then the macroscopic results will be compared with the experimental results.

### FSI in the microscopic model

**Fig. 4:**
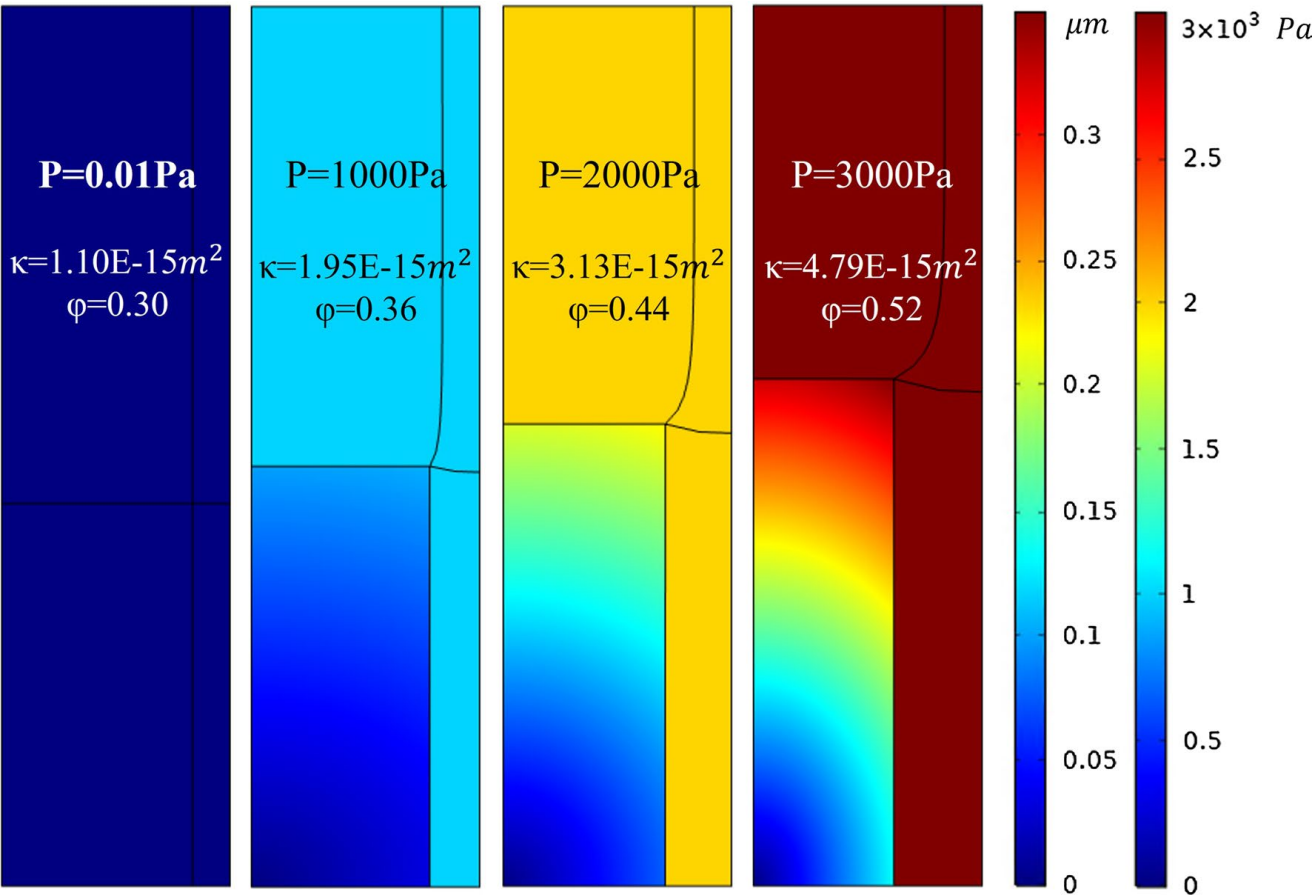
Results of FSI in microscopic model with different pressures

Transient two-way fully coupled FSI method was used in the microscopic model. It can be seen from Fig. 4 that with the increment of local pressure, the axon is gradually compressed in the radial direction, which gives more space for the IF, thus increasing the local permeability and porosity. From the pressure distribution of IF, it can be seen that the pressure is uniform along the flow path, which meets the requirements of Sect. 2.6 (2).

### Effects of microscopic FSI on the heterogeneity of permeability and porosity

**Fig. 5:**
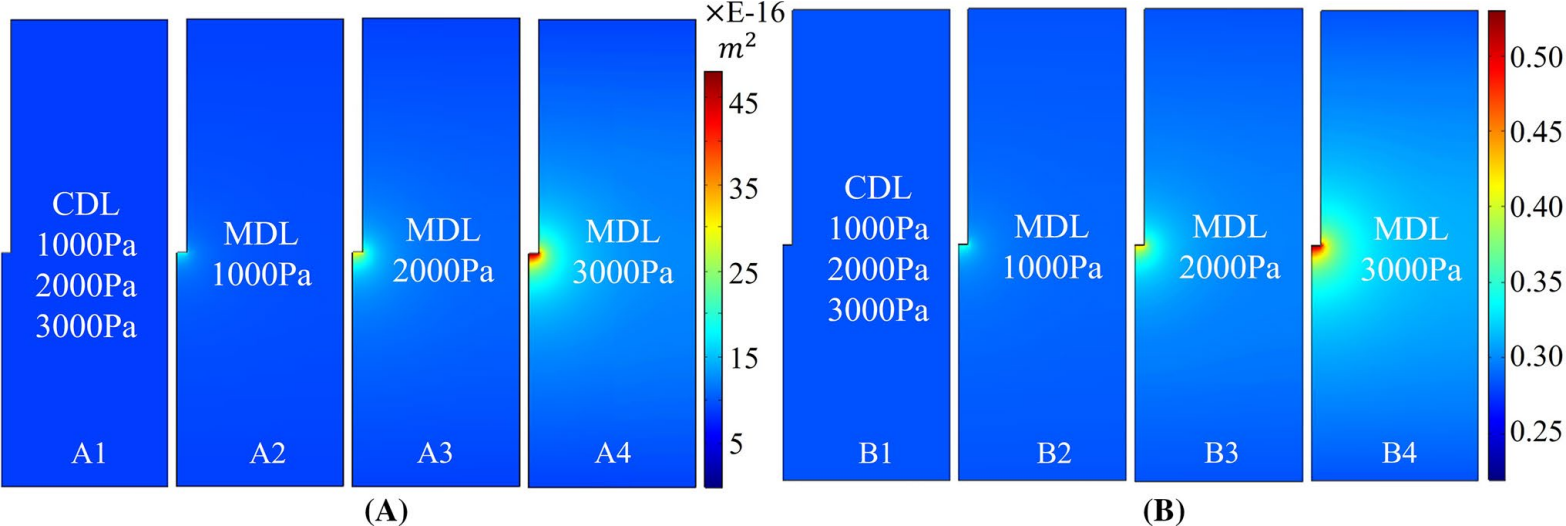
Distribution of local permeability and porosity in macroscopic model with different mathematical models and different infusion pressures. **a** Permeability and **b** porosity

Figure 5 shows that microscopic FSI leads to a heterogeneous behaviour of brain’s permeability and porosity. When classical Darcy’s law (CDL) was employed, which assumes that the microstructure is rigid, permeability and porosity of the tissue were uniform and homogeneous in the soft porous domain no matter what the local pressure was, as shown in Fig. 5a1, b1. This is obviously incongruous with the experimental observation (Jamal et al. 2021).

In contrast, the heterogeneous distribution of tissue permeability and porosity can be predicted using the newly developed microstructurally based Darcy’s law (MDL). From Fig. 5a2–a4, b2–b4, it can be found that local permeability and porosity were higher in the region which was close to the infusion point, where the maximum pressure was achieved. When the infusion pressure was low, the tissue porosity and permeability would be almost homogeneous, as shown in Fig. 5a2, b2.

### Effects of microscopic FSI on the global distribution of pressure and drug infusion

**Fig. 6:**
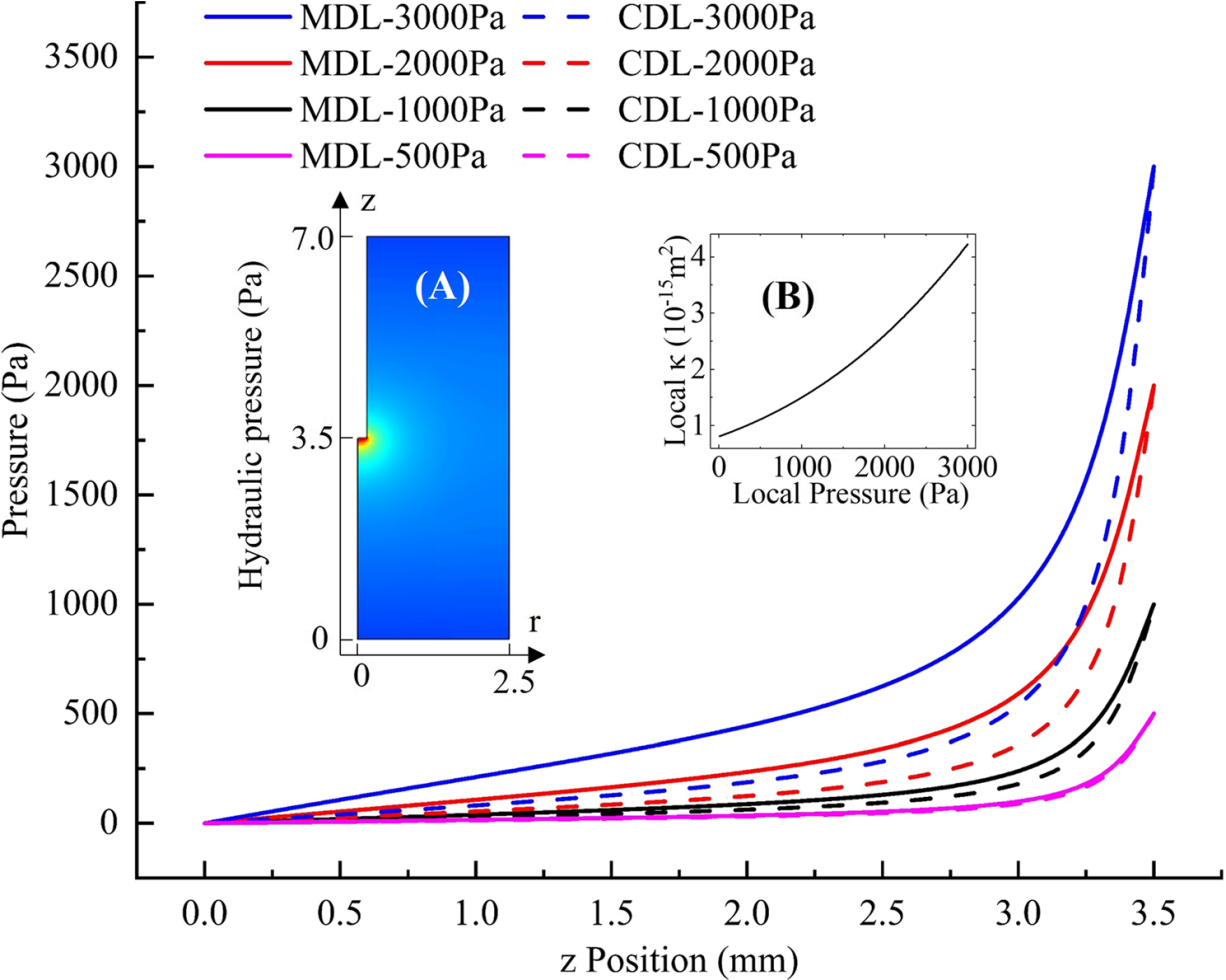
Comparison of the pressure distribution along the infusion axis between the results of MDL and CDL with different infusion pressures. **a** Hydraulic pressure distribution in the tissue. **b **Local pressure–local permeability relationship of the microscopic model.

**Fig. 7:**
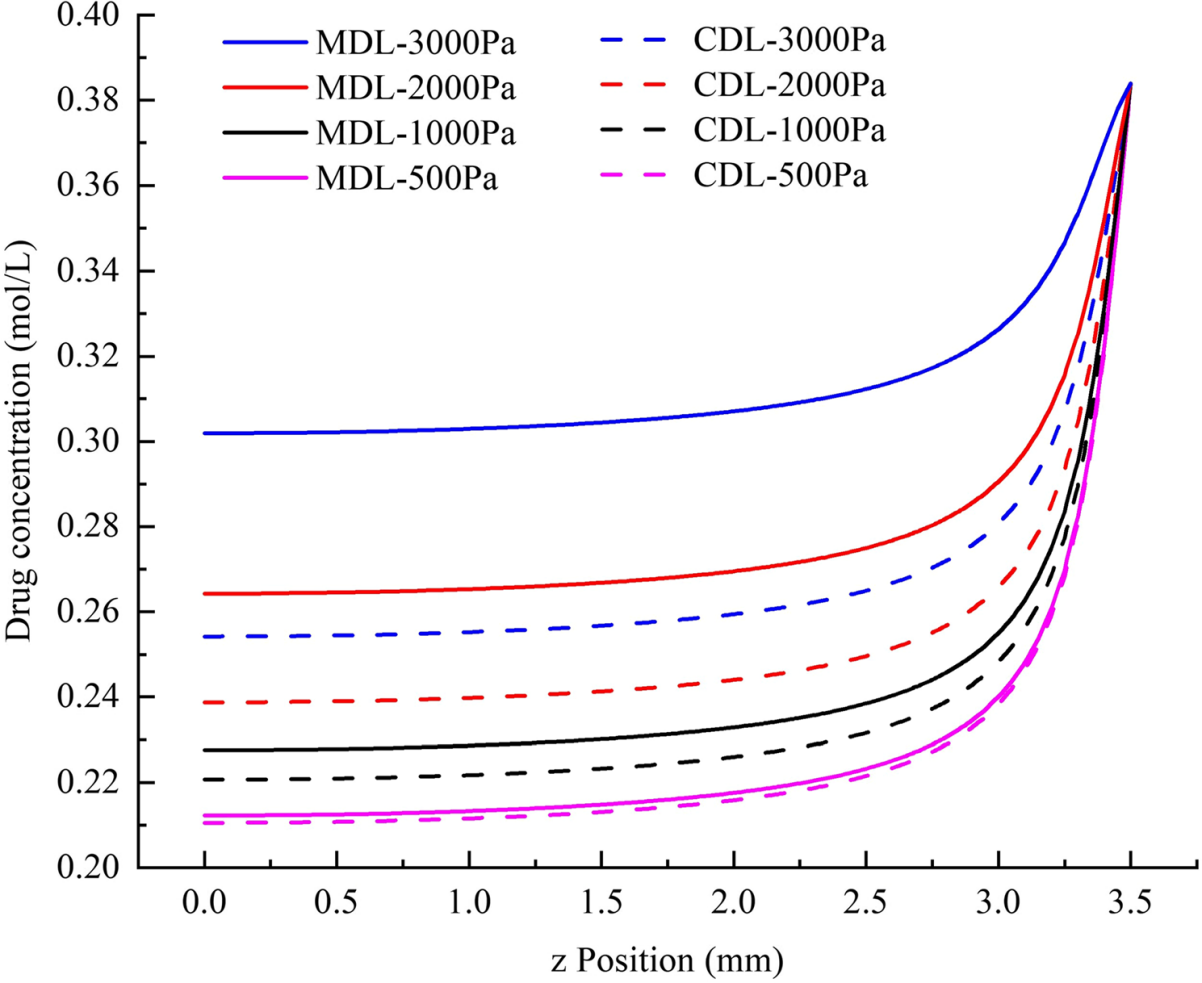
Comparison between the results obtained using MDL and CDL in terms of drug concentration distribution along the infusion axis with different infusion pressures

Brain microstructure will certainly deform with the change of local hydraulic pressure, thus altering the local permeability and porosity, and leading to the heterogeneous response of the whole domain. What really matters, in fact, is to know whether and how this local FSI can affect the global distribution of hydraulic pressure and drug concentration in the tissue. The answers have been quantitatively obtained and are shown in Figs. 6 and 7. There are two major points worth considering regarding pressure distribution in Fig. 6:

1. Hydraulic pressure declines more slowly along the infusion direction (from (0, 3.5) to (0,0) in Fig. 6a) if MDL is adopted, which is reflected in the slope differences between the solid lines and the corresponding dashed lines in Fig. 6. The reason is that the local porosity and permeability at a specific position will be higher if local FSI is considered (see Fig. 4), thus the pressure gradient will be smaller according to Darcy’s law.
2. The effect of local FSI on global distribution of hydraulic pressure and pressure gradient is highly dependent on the infusion pressure. When the infusion pressure is high, the differences between the pressures calculated by CDL and MDL are bigger, since the relationship between local pressure and local permeability is nonlinear, as shown in Fig. 6b. When local pressure is higher, local permeability increases faster with the increase in local pressure. Consequently, the global pressure decreases in a slower manner because pressure drop is negatively correlated to permeability according to Darcy’s law (Eq. 1). In summary, the higher infusion pressure is, the greater influence local FSI will have on the hydraulic pressure distribution. When infusion pressure is no higher than approximately 500 Pa, local FSI has very limited effect and could be neglected.

To understand how local FSI affects drug distribution in brain tissue, a quantitative study was carried out. Assuming that 5-Fluorouracil (5-FU) solution, a cytotoxic chemotherapy medication used to treat cancer (Longley et al. 2003), was infused from the inlet boundary with a constant concentration of 50 mg/mL (Fluorouracil dosing 2014). In the outlet boundaries, the concentration was 0 (see Fig. 3c). By solving diffusion–convection–reaction equation in the porous medium, as described in "Appendix C", the drug concentration distribution in the macroscopic model was obtained using both CDL and MDL to describe fluid flow; the results obtained are shown in Fig. 7.

Similar to the results obtained for pressure distribution, drug concentration decreases more slowly along the infusion axis if local FSI is considered. The effect of local FSI on drug distribution is also more significant with a higher infusion pressure.

When the infusion pressure was 500 Pa, the difference between the results of CDL and MDL was very limited; but when the infusion pressure reached 3000 Pa, an intermediate level of infusion pressure in CED trials (Hunt Bobo et al. 1994; Raghavan et al. 2006), the difference of drug concentration was shown to be as high as 16%. This implies that if local FSI is not considered when providing preoperative suggestions for CED operations, the drug concentration in brain tissue will be significantly underestimated. As a result, the actual injected dose may exceed the recommended value, which may be potentially dangerous, especially for cytotoxic chemotherapy (Aston et al. 2017).

### Direct comparison with experimental evidence

To quantify the relationship between infusion pressure and hydraulic permeability, in vitro experiments with 50 samples from ovine brains were conducted to measure the hydraulic permeability of the tissues with different infusion pressures (see Fig. 2a). Here, we validate the mathematical model with the experimental results, and the cases in which infusion direction is parallel to the axons’ orientation were used in this paper.

**Fig. 8:**
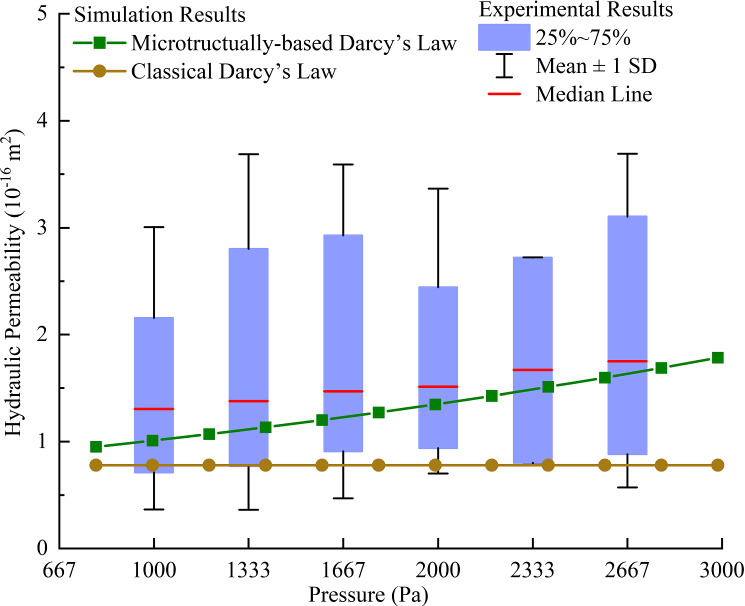
Comparison of the pressure–permeability relationship between numerical results and experimental results

The results of hydraulic permeability of the different samples under specific infusion pressure are drawn to be the box chart in Fig. 8. The blue boxes contain Q1–Q3 percentile of the individual group of data points and the median value is labelled with red line. According to Fig. 8, the simulation results agree well with the experimental data, as they share the similar trends, and all of the simulation result points fall within the Q1–Q3 boxes of the experimental data.

However, there are two points worth of deeper discussions. One is about the difference on the magnitudes. There are in fact some details, such as the proteins and fibres in the ECM of the microscopic model were ignored. These very small components serve as obstacles in the flow path and prevent the fluid from passing by, thus decreasing the hydraulic permeability. It means that the calculated permeability should be higher than the tested value, whereas the results in Fig. 8 show the opposite. The other one is about the difference on the slope of the pressure–permeability relationships. The predicted curve has a larger slope than that of the tested results. This might imply that mathematical model is imperfect due to its inability to deal with high infusion pressures (and deformations) and suggest that the reconstructed microstructural model is more sensitive to the pressure than the tested samples.

**Fig. 9:**
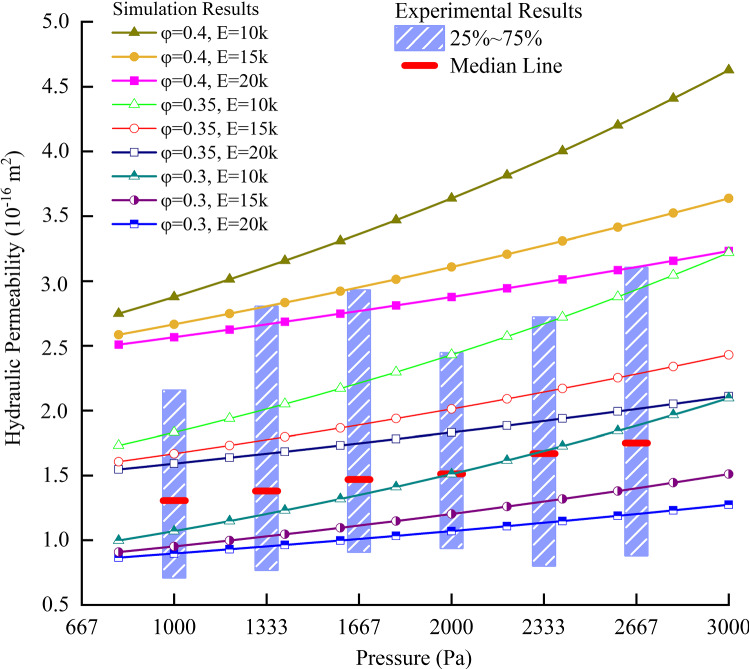
Results of numerical experiments based on the multiscale model

In order to explore the reasons behind these problems, some more numerical experiments were done based on the multiscale model. Specifically, Young’s modulus of the axon and tissue porosity were chosen to do parametric studies to investigate how these two parameters can affect the pressure–permeability relationship. According to the discussion above, porosity should be bigger than 0.3 if higher permeability needs to be obtained, so porosity of 0.3, 0.35, and 0.4 was considered. In addition, Young’s modulus of 10 kPa, 15 kPa, and 20 kPa, which are close to 12 kPa and within the range in published database (Bernal et al. 2007; Spedden et al. 2012; Ouyang et al. 2013; Chen et al. 2019) were adopted. It can be seen from the results in Fig. 9 that both parameters have significant effects on the pressure–permeability relationship, but their ways of influence are quite different.

Increasing tissue porosity can significantly increase the magnitude of permeability; however, this has limited effect on the slope of the pressure–permeability curves. This is because the slope of pressure–permeability curves depends on how much the change of local pressure affects the local microstructure (or porosity), but not on the overall porosity itself. The stiffness of the axon has effects on both the magnitude of permeability and the slope of the pressure–permeability relationship. High Young’s modulus always leads to low permeability and less effect of pressure on permeability. The reason for this is that when the structure has a larger Young’s modulus, it undergoes less deformation, which prevents the gaps from expanding and thereby limits the increase in local permeability with pressure. Therefore, stiffer axons result in low permeability when local FSI is considered. In addition, when the Young’s modulus of the axon is higher, the same increment of local pressure produces smaller axons’ deformation. As a result, the permeability will increase more slowly with the increase in local pressure.

The above analyses indicate that the tissue porosity and Young’s modulus of the axons are the key parameters that affect the prediction accuracy of this mathematical model. In fact, the assumption of macroscopic isotropy should also cause the overestimation of the pressure–permeability slope. The perpendicular component of permeability is less sensitive to the local pressure than the parallel component according to our experiments; while the experimental results came from the whole permeability tensor, the simulation assumed isotropic permeability that contains only parallel component.

## Discussion

Understanding how fluid flows in brain tissue is very important to not only maintain brain health but also treat brain diseases. However, direct adoption of CDL without considering local microstructural FSI effects may lead to large errors when estimating the realistic transport properties of the tissue. This is because Darcy’s law in its original form is unable to consider the change of permeability and porosity caused by pressure-driven microstructural deformation. The multiscale model developed in this paper couples Darcy’s law at macroscopic and FSI at microscopic, thus being able to consider the microstructurally driven heterogeneous response of brain tissue, overcomes this limitation.

Considering biomedical applications, the multiscale model built and validated in this work could be adopted to increase the proficiency of clinical treatments for brain diseases by visualizing the flow and concentration field in the brain with a higher accuracy. According to our simulations and in accordance with the limited existing experimental evidence, the variations of background intracranial pressure, as described in the introduction section, might have been significant enough to change the permeability and porosity of brain tissue, which would alter the flow path of the infused drug. This effect is more considerable if the infusion pressure is added. By employing this model, a more accurate preoperative prediction of the drug flow path and concentration distribution could be obtained. Furthermore, as CDL would underestimate both hydraulic pressure and drug concentration in brain tissue when high infusion pressure is applied, adoptions of the newly developed model would provide relatively safer suggestions for preoperative planning. This multiscale framework could also be used or serve as a reference for the development of models for other soft tissues and procedures where fluid pressure plays a major role in the tissue response.

The present multiscale model also established a direct relationship between hydrostatic pressure and hydraulic permeability as a function of the microstructural deformation of brain tissue. The damage to the infusion site due to needle insertion and high local pressure is still one of the major drawbacks of CED treatment (Fattahi et al. 2014). The infusion pressure must be low enough to avoid damage to the neuronal cells but also needs to be high enough to ensure the penetration. Therefore, there should be an optimal value for the infusion pressure. The newly developed modelling method provides a potentially practical tool to find the optimal value of infusion pressure by accurately modelling the interplay between microscopic deformations and macroscopic pressure distribution.

In terms of computational simulations of drug delivery in biological tissues, the use of the microscopic FSI has prompted some important considerations. (i) Hydraulic permeability of biological tissues is very sensitive to porosity. With a small increase in porosity, for example 0.1, the value of tissue permeability can change by a factor of 2, as shown in Fig. 9. This means that great attentions should be paid to the porosity when dealing with fluid flow in biological tissues. The growing evidence of volume change of the extracellular space during the sleep–wake cycle in the mouse cortex (Xie et al. 2013; Ding et al. 2016) is quite important (up to 50%). The present study suggests that such a change of the ECS will have a strong impact on the drug delivery and should be considered for chronic intermittent drug delivery. (ii) Deformation of the microstructure cannot be ignored in the calculation of fluid field in biological tissues, especially when the pressure is relatively high, and the tissue is extremely soft. For example, for Young’s moduli of the axon in the order of 10 kPa, the tissue permeability could be doubled when the infusion pressure is increased from 1000 to 3000 Pa, and this trend is also increasing with the increment of pressure. Therefore, to get more precise predictions of drug delivery in the brain, experimental characterizations of Young’s modulus of the axons and porosity of the tissue are of great importance.

In fact, the results of our model not only help to understand the effect of Young’s modulus of the axon and porosity of the tissue on brain’s behaviour during drug infusion, but also provide insights into numerical characterization of these two parameters. The mechanical properties of axons are of great importance, especially in the investigations of axonal injury (Cloots et al. 2012; Finan et al. 2017), and the mechanism of axon growth and regeneration (Koser et al. 2016). Therefore, many experimental (Bernal et al. 2007; O’Toole et al. 2008; Spedden et al. 2012; Montanino et al. 2019) and computational (Karami et al. 2009; Cloots et al. 2012; Ouyang et al. 2013; Chen et al. 2019) studies have been done to characterize the mechanical properties of axons. However, from the existing database, the value of Young’s modulus of axons is reported to vary in a broad range from 100 Pa to 0.1 MPa (Bernal et al. 2007). Similarly, it is hard to measure the exact porosity of brain tissue due to the complexity of experimental environments and conditions. Currently, experiments show that the distance between axons, which in turn determine porosity, is in the range from 38 nm to 3.2 µm (Thorne and Nicholson 2006; Tønnesen et al. 2018) and the porosity is less than 0.3 (Vidotto et al. 2019). Using the results from our model presented in Fig. 9, we might be able to determine the approximate value of these two important parameters by combing the mathematical model established in this study and experimental tests in (Jamal et al. 2021). In this study, for example, when the apparent porosity of the tissue and Young’s modulus of the axons are about 0.32 and 20,000 Pa, respectively, they can produce the best match of the results with the experiments.

Although the results obtained by the present multiscale model agree well with the experimental tests in the specific conditions tested (low pressure and infusion rates), it is still worth discussing the simplifications adopted in this model, which provide useful information about further improvements and potential extensions of this method. (i) The geometry of the microscopic model, albeit using realistic statistical data, is idealized. For example, the macromolecules in the ECM that obstruct the IF flow, were not modelled. However, the measured viscosity of IF instead the viscosity of drug fluid (closer to water) was used in the modelling, as shown in Table 1. This enabled to partly include the effects of these components in the measured value of viscosity. Additionally, this paper focuses on the impact of neural cells on the solute transport without considering the important roles played by the glial and accessory cells. In particular, astrocytes have specific bidirectional water channels (aquaporin4) on their membrane and can therefore change their volume and affect local transport properties (Wilcock et al. 2009). Furthermore, the geometry of axon was also simplified. The mathematical model in this paper can be combined with realistic brain microstructure reconstructed from microscopic images (Bernardini et al. 2021; Vidotto et al. 2021) to further improve the model accuracy. (ii) The anisotropy of the brain tissue in the macroscopic model was not explicitly described and both the tissue permeability and drug diffusivity were treated as isotropic. The fluid flow is different when pressure gradients are applied parallel or perpendicular to the axons. Our experimental study has shown that infusion pressure has less effect on the permeability when fluid is infused perpendicular to the axons (Jamal et al. 2021). (iii) One of the simplifications used in this work was the choice of linear elasticity for the description of the constitutive behaviour of axons. Overall, since the work presented in this paper is focused on the establishment of the new multiscale modelling framework and the exploration of its medical applications, only one single axon orientation and a linear elastic material model were used. However, the method can be easily extended to include anisotropic and hyperelastic/viscoelastic features, and these will be addressed in future contributions. In our future work, a 3D microstructurally accurate model will be constructed to provide a more systematic database for the results and an ideal starting point for the simulations. Through its use, both the issues of tissue anisotropy and pressure gradient can be considered and addressed.

## Conclusions

In this paper, we developed a multiscale modelling framework that accounts for the microstructurally driven heterogeneity of the local variation of permeability and porosity in the brain tissue. Simulation results agreed well with the experimental data obtained when studying the link between permeability and infusion pressure in ovine brain tissue, which demonstrates the potential applicability of this multiscale model to capture the dependence of drug infusion reach and dynamics on microstructural features. As results show that both hydraulic pressure and drug concentration in the brain would be significantly underestimated by CDL, the importance of the newly developed method is highlighted. It is an essential step towards providing accurate preoperative predictions for CED treatment and an efficient modelling tool for the development of new drugs for brain disorders and medical equipment for CED surgery. Furthermore, the developed multiscale model provides a new framework which can be used to simulate the pressure-dependent response of soft porous material.

## Figures and Tables

**Fig. 10:**
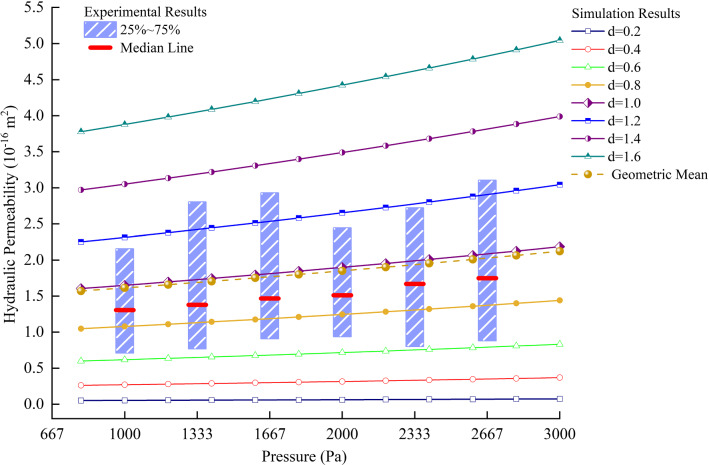
Effect of axon’s diameter on the apparent permeability

**Fig. 11:**
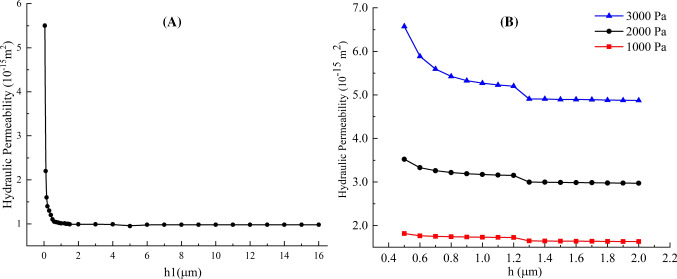
**a** Relationship between effective length of the microscopic model and its hydraulic permeability. **b** Relationship between elongated length of computational domain and the calculated hydraulic permeability

**Fig. 12:**
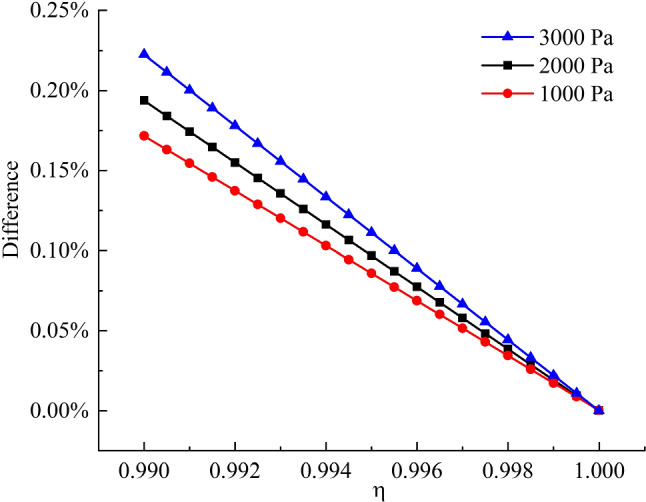
Relationship between *η* and the calculation difference of hydraulic permeability

## Appendix A: Determination of representative axon diameter

Axons’ diameter is not uniform but in a range with the mean value of around 1 $\upmu {\rm m}$ (Bernardini et al. 2021). Therefore, in the present microscopic model, the diameter of the axon was assumed to be 1 $\upmu {\rm m}$. To validate this assumption, a further study was done to help understand the effect of axons’ diameter on the apparent permeability calculated by this multiscale model. 8 microscopic models with the axon’s diameters ranging from 0.2 $\upmu {\rm m}$ to 1.6 $\upmu {\rm m}$ with an interval of 0.2 $\upmu {\rm m}$ were built. The mean cross-sectional area of the 8 models is the same as the cross-sectional area of the 1 $\upmu {\rm m}$ axons model. The porosity of all the models was 0.35 and the Young’s modulus of the axons was set to be 20 kPa. Figure 10 presents the results of the 8 models regarding the relationship between hydraulic pressure and the apparent permeability of the macroscopic mode, which suggests that the size effect of the axon’s diameter may be non-negligible in the multiscale model. However, if we calculate the mean value of the 8 model’s permeability and compare this mean value with the permeability of the 1 $\upmu {\rm m}$ axons model, it is found that the two results match well, as shown in Fig. 10 by comparing the curves labelled as *d* = 1.0 $\upmu {\rm m}$ and the results given by taking the geometric mean of all simulations. This means that if the representative axon has the same cross-sectional area as the mean value of all axons’ cross-sectional areas, permeability of a larger domain including different axons of different diameters could be obtained by using this representative axon model.

## Appendix B: Sensitivity study

### (1) h1

In the sensitive study on h1, the values of (0.01 μm, 0.01 μm, 0.09 μm), (0.1 μm, 0.1 μm, 0.9 μm), (1 μm, 1 μm, 15 μm) were adopted since the average length of axons is measured as 15 μm (Abdollahzadeh et al. 2019). The numbers in the brackets mean (start number, increase step, finial number). The relationship between h1 and hydraulic permeability of the microscopic model is shown in Fig. 11a, which indicates that when h1 > 1 μm, h1 will have no obvious effect on hydraulic permeability of the microscopic model. Therefore, h1 was set as 1 μm in this study.

### (2) h2

In terms of h2, the values of (0.5 μm, 0.1 μm, 2 μm) were chosen to do sensitive study. The relationship between h2 and calculated permeability is shown in Fig. 11b. When h is bigger than 1.3, the effect of h on the calculated permeability can also be neglected. Thus, h2 was set as 1.3.

### (3) *η*

The aim of adopting *η* is to make the pressure in the microscopic model to be nearly uniform, so the value of *η* should be better close to 1. Therefore, the values of (0.990, 0.001, 0.999) were chosen to conduct sensitive study for *η*. Figure 12 presents the relative difference between the calculated permeability of the specific *η* and that of when *η* = 1, in a formula form:

B.1 $$\begin{array}{c}Difference=\frac{{k|}_{\eta =1} \ \ - \ {k|}_{\eta }}{{k|}_{\eta =1}}\end{array}$$

where $k$ denotes the calculated local permeability.

It can be found that when *η* > 0.999, pressure drop in the fluid domain is small enough to be neglected. Therefore, *η* was set as 0.999 in the microscopic model.

## Appendix C: Calculation of drug distribution

Diffusion–convection–reaction equations, as given in Eqs. (C.1–C.3), were solved to obtain drug concentration distribution in brain tissue.

C.1 $$\begin{array}{c}\phi \frac{\partial c}{\partial t }+\nabla \cdot \left({\mathbf{v}}_{\mathbf{D}}c\right)-D{\nabla }^{2}c=0\end{array}$$

where $\phi$ is the porosity of tissue, $c$ represents the drug concentration, $D$ is the tensor of effective diffusivity of the drug, and ${\mathbf{v}}_{{\varvec{D}}}$ denotes the macroscopic fluid velocity given by Darcy’s law:

C.2 $$\begin{array}{c}{v}_{\mathrm{D}}=-\frac{k}{\mu }\nabla P.\end{array}$$

To consider the concentration-dependent 5-FU elimination, Michaelis–Menten kinetics (Srinivasan 2021) was adopted and modelled as:

C.3 $$\begin{array}{c}D\nabla c=-{\mathrm{V}}_{\mathrm{max}}\frac{c}{{K}_{m}+c}\end{array}$$

where ${\mathrm{V}}_{\mathrm{max}}$ is the maximum elimination rate, and ${K}_{m}$ is the Michaelis constant, which is numerically equal to the substrate concentration at which the reaction rate is half of ${\mathrm{V}}_{\mathrm{max}}$.

The parameters that were used in this case study are listed in Table 2. It is worth mentioning that the effective diffusion coefficient of drug molecules is also anisotropic in WM (Yuan et al. 2022). However, this anisotropic diffusivity of 5-FU in the WM is not available in the open literature, so the effective diffusion coefficient was also assumed to be isotropic and porosity independent.

**Table 2:** Parameters used in the case study

Parameters	Unit	Value	Description	Source
${c}_{0}$	mg/mL	50	Concentration of 5-FU solution at inlet boundary	Fluorouracil dosing (2014)
${M}_{5-FU}$	g/mol	130.078	Molecular weight of 5-FU	Fluorouracil dosing (2014)
$D$	${\mathrm{m}}^{2}/\mathrm{s}$	$1.2\times {10}^{-9}$	Diffusion coefficient of 5-FU	Saltzman and Radomsky (1991)
${\mathrm{V}}_{\mathrm{max}}$	$\mathrm{mol}/L/\mathrm{s}$	$5.9\times {10}^{-7}$	Maximum elimination rate of 5-FU	Spoelstra et al. (1991)
${K}_{m}$	$\mathrm{mol}/\mathrm{L}$	$16\times {10}^{-6}$	Michaelis constant	Spoelstra et al. (1991)
